# Genome sequence of *Microbacterium* phage Casino

**DOI:** 10.1128/mra.00018-25

**Published:** 2025-06-09

**Authors:** Jennifer H. Arca, Gillian A. Bradley, Lola M. Gantenbein, Landon H. Schumaker, Raelynn A. Amasol-Tanoura, Avery C. Catlin, Saige A. Edwards, Sophia F. Faria, Maximilliano Fuentes-Ayala, Alexis M. Haiges, Hailey E. Hart, Ayden K. Herrera, Paisley J. Karlin, Ian T. Rife, Nana Sannomiya, Katie N. Thai, Emily J. Velasquez, Cori B. Williams, Hannah E. Moon, Megan L. Porter

**Affiliations:** 1 School of Life Sciences, University of Hawaiʻi at Mānoa, Honolulu, Hawaiʻi, USA; Loyola University Chicago, Chicago, Illinois, USA

**Keywords:** actinobacteriophage, genomes, *Microbacterium*

## Abstract

We present the genome of Casino, a lytic actinobacteriophage assigned to the EM1 subcluster. Casino infects *Microbacterium foliorum* NRRL B-24224 and was isolated from a soil sample in Storrs, Connecticut. The genome is 53,667 bp in length with 48 annotated genes and a GC content of 64.8%.

## ANNOUNCEMENT

Bacteriophages are bacteria-infecting viruses that establish parasitic relationships with their hosts through lytic or lysogenic cycles. As bacteriophages are developed as treatments for bacterial infections, the vast diversity of undiscovered phages represents untapped potential (1). We present the genome of the actinobacteriophage Casino, an EM1 subcluster phage that infects *Microbacterium foliorum* strain NRRL B-24224.

Casino was isolated from a residential soil sample in Storrs, Connecticut, in 2022 (GPS coordinates: 41.78307 N, 72.24083). The sample was isolated at 21°C, using standard methods (2). The soil sample was washed in a PYCa medium and filtered through a 0.2 μm filter. The filtrate was plated on PYCa top agar containing *M. foliorum* NRRL B-24224 and incubated at 30°C until clear round plaques formed (~24–48 hrs). Multiple rounds of plating, including plaque assays followed by spot tests, were conducted to prepare a purified phage lysate.

Genomic DNA was extracted from the lysate using the Promega Wizard DNA Clean-up Kit and prepared for sequencing using the NEB Ultra II Library Kit, before being sequenced using Illumina Shotgun Sequencing (MiSeq, v3 reagents), generating 382,391 150-base single-end reads. Raw reads were assembled in Newbler v2 (Roche), resulting in a single genomic contig with 1012-fold coverage. This genome was annotated using the Phage Evidence Collection and Annotation Network (PECAAN) v20240320 (https://discover.kbrinsgd.org). Start sites were refined using Phamerator v3 (3), Starterator v1.2 (http://phages.wustl.edu/starterator), Glimmer v3.02B (4), and GeneMark v4.28 (5). Potential functions for predicted protein-coding genes were determined using NCBI BLASTp v2.13.0+ (nonredundant database) (6) and HHpred (PDB mmcif70, NCBI conserved domain, SCOPe70, and Pfam databases) (7). Additionally, putative membrane proteins were detected with TMHMM v1.0.24 (8). The default parameters were used for all software.

The genome is circularly permuted with 53,667 bp and a GC content of 64.80%. Casino is predicted to have 48 genes, 16 (33.3%) of which have been assigned a putative function, while the remaining 32 genes (66.7%) encode hypothetical proteins with unknown functions. No tRNAs were identified with ARAGORN v1.2.41 (9) and tRNAscan-SE v2.0.9 (10). Based on gene content similarity of at least 35% to phages in the Actinobacteriophage database (11, 12), Casino was assigned to phage cluster EM1. As with other cluster EM1 phages, genes encoded within the first and second halves of the genome are transcribed in opposite directions. Identifiable DNA metabolism functions encoded within the first half of the genome include Cas4 exonuclease, DNA polymerase I, and DNA helicase, whereas structure, assembly, and lysis functions are encoded within the second half of the genome and include terminase, portal, and tail proteins, as well as Lysin A. Consistent with the podovirus morphology of previously characterized cluster EM1 phages, no gene encoding a tape measure protein involved in the biogenesis of tails for siphoviruses and myoviruses could be identified.

## Data Availability

Casino sequence data is available at GenBank with Accession No. PP978794 and Sequence Read Archive (SRA) No. SRX24892112.
